# IL-17RA in Non-Hematopoietic Cells Controls CXCL-1 and 5 Critical to Recruit Neutrophils to the Lung of Mycobacteria-Infected Mice during the Adaptive Immune Response

**DOI:** 10.1371/journal.pone.0149455

**Published:** 2016-02-12

**Authors:** Robin Lombard, Emilie Doz, Florence Carreras, Mathieu Epardaud, Yves Le Vern, Dominique Buzoni-Gatel, Nathalie Winter

**Affiliations:** INRA, Université de Tours, UMR 1282, Infectiologie et Santé Publique, Nouzilly, France; Public Health Research Institute at RBHS, UNITED STATES

## Abstract

During chronic infection with *Mycobacterium tuberculosis* (Mtb), bacilli multiplication is constrained within lung granulomas until excessive inflammation destroys the lung. Neutrophils are recruited early and participate in granuloma formation, but excessive neutrophilia exacerbates the tuberculosis disease. Neutrophils thus appear as potential targets for therapeutic interventions, especially in patients for whom no antibiotic treatment is possible. Signals that regulate neutrophil recruitment to the lung during mycobacterial infection need to be better understood. We demonstrated here, in the mouse model, that neutrophils were recruited to the lung in two waves after intranasal infection with virulent Mtb or the live attenuated vaccine strain Bacillus Calmette Guérin (BCG). A first wave of neutrophils was swiftly recruited, followed by a subsequent adaptive wave that reached the lung together with IFN-γ- and IL-17A-producing T cells. Interestingly, the second neutrophil wave did not participate to mycobacteria control in the lung and established contacts with T cells. The adaptive wave was critically dependent on the expression of IL-17RA, the receptor for IL-17A, expressed in non-hematopoietic cells. In absence of this receptor, curtailed CXCL-1 and 5 production in the lung restrained neutrophil recruitment. CXCL-1 and 5 instillation reconstituted lung neutrophil recruitment in BCG-infected IL17RA-/- mice.

## Introduction

Following exposure to virulent *Mycobacterium tuberculosis* (Mtb), one of the three leading infectious cause of human mortality [1], a large number of individuals do not show evidence of T-cell sensitization suggesting that innate mechanisms in the lung may clear infection [2]. In others, the adaptive immune response, characterized by a delayed hypersensitivity reaction to tuberculin, is initiated. However, this is generally not enough to eradicate all bacilli and most people remain latently infected with Mtb. The estimated latent tuberculosis (TB) reservoir currently corresponds to about one third of the world population [3]. Vaccination with *M*. *bovis* Bacillus Calmette Guérin (BCG), a live attenuated strain, induces a strong and long-lasting immune response. However, BCG provides high levels of protection only against the most severe forms of TB and, despite broad vaccination coverage, BCG is unable to control global pandemics of TB [4]. The WHO has declared the fight against TB to be a global priority. In latently infected individuals, CD4 and CD8 T and B cells that are recruited to the lung together with innate cells, form a specific multicellular structure, the granuloma [5]. Excessive inflammation within the granuloma leads to caseification and lung tissue destruction. The roles of macrophages in mycobacterial killing and evasion, and of dendritic cells in linking innate and adaptive responses to mycobacteria are well established [6]. The role played by neutrophils is more debated. They are among the first cells to respond to mycobacterial infection and participate in the onset of adaptive immunity [7, 8] and granuloma formation [9]. However, chronic neutrophilia is involved in TB physiopathology, although the mechanisms underlying neutrophil accumulation long after primary infection are not entirely clear [10–12].

IL-17 cytokines play an important role in inflammation. The best characterized member of this large family is IL-17A. IL-17F is closely related to IL-17A and these two molecules can form heterodimers with different effects on the fine-tuning of the inflammatory response depending on the pathological context [13]. IL-17 cytokines signal through receptors of the IL-17R family consisting of five subunits, which can assemble in different combinations to form diverse functional receptors. The IL-17RA subunit is common to several receptors used by at least four ligands containing IL-17A or F proteins [14]. IL-17 receptors mediate signaling through pathways generally associated with innate immunity and they connect the innate and adaptive arms of the immune response [14]. IL-17RA is expressed ubiquitously, and particularly in non hematopoietic epithelial cells, endothelial cells and fibroblasts [14]. In response to mycobacterial infection, IL-17A is produced principally by CD4^+^ CD8^-^ αβ^+^ T cells, which are also known as Th17 cells [15], and ϒδ+ T cells [16, 17]. Vaccine-induced Th17 cells favor the recruitment of protective Th1 cells in response to Mtb infection [15], IL-17A contributes to the formation of a mature granuloma [17, 18] and is required to constrain multiplication of Mtb clinical isolates [19] demonstrating beneficial effects. However, IL-17A is also detrimental because its unrestricted production leads to lung tissue destruction [10].

We investigated how neutrophils were recruited to the lung in mice inoculated by the intranasal (i.n.) route with high dose of live attenuated BCG or low dose of virulent Mtb. While BCG multiplication in the lung was controlled by the adaptive response, Mtb was not. In both situations we observed that, in addition to neutrophils recruited early in infection, a second adaptive wave of neutrophils was recruited to the lung, together with T cells. IL-17RA expressed by non-hematopoietic cells, was critically involved in the adaptive wave of neutrophil recruitment. In absence of this receptor, even though CXCL-2 was produced, CXCL-1 and 5 production in the lung was curtailed and neutrophils were not recruited. CXCL-1 and 5 instillation restored lung recruitment to the lung in IL-17RA-/- BCG-infected mice.

## Materials and Methods

### Ethics statement and mouse treatments

Experimental protocols complied with French law (Décret: 2001–464 29/05/01) and EEC regulations (86/609/CEE) for the care and use of laboratory animals and were carried out under Authorization for Experimentation on Laboratory Animals Number B-37-201. Our animal protocol (number 2012-06-14) was approved by the “Val de Loire” Ethics Committee for Animal Experimentation (CEEA VdL) and was registered with the French National Committee for Animal Experimentation.

Six- to eight-week-old C57BL/6 male mice were obtained from SAS Janvier (Le Genest Saint Isle—France); IL-17RA-/- mice originally produced by Ye and coworkers [20] were reared in the INRA specific pathogen-free resident animal facility. Mice, anesthetized by i.p. injection of ketamine/xylasine cocktail, received 5x10^6^ CFU of BCG or 10^3^ CFU of Mtb under 20 **μ**l in each nostril. To deplete the neutrophil adaptive wave, mice were i.p. injected on day 17, 19 and 21 following BCG or Mtb inoculation with 200 μg of NIMPR14 mAb [21] which specifically depletes Ly-6G+ neutrophils, control mice were injected with the same quantity of IgG2b Ab (both produced in the laboratory).We produced hematopoietic chimeras, by injecting 7x10^6^ BM cells from either WT or IL-17RA-/- donor mice intravenously into WT or IL-17RA-/- mice. Six weeks later, we checked the genotype of the donor BM, by performing PCR on blood cells.

CXCL-1 and 5 chemokines (R&D systems, Minneapolis, MN, USA) were mixed in PBS and instilled i.n. to mice at day 20 μl after BCG infection. Each mouse received 100 μg of each chemokine under 20 μl in each nostril. Control mice received equivalent volume of PBS.

### Bacterial strains and CFU determination

Wild-type BCG strain 1173P2 Pasteur [22] and recombinant Myc 409 strains expressing EGFP [7] were grown in Beck-Proskauer medium, supplemented with hygromycin (50 μg/ml) for Myc 409. The Mtb strain laboratory adapted strain from human origin H37Rv was cultured in 7H9 medium supplemented with ADC (5% BSA fraction V, 2% dextrose, 0.003% beef catalase and 0.85% NaCl; BD Microbiology Systems, San Jose, CA, USA). The fast growing non pathogenic species *M*. *smegmatis* was grown in LB medium (Becton Dickinson, USA) Bacteria were harvested at mid-exponential growth phase and frozen at -70°C. CFU were counted after plating dilutions on Middlebrook 7H11 agar supplemented with OADC (ADC supplemented with 0.005% oleic acid, BD Microbiology Systems). For the preparation of heat-killed (HK) BCG, bacilli suspended in PBS were heated for 30 min at 80°C. For lung CFU determination, tissues were disrupted with ceramic beads (Lysing Matrix D, MP Biomedicals Europe, Illkirch, France) in PBS, in a Ribolyser apparatus (Fastprep-24, MP Biomedicals), in accordance with the manufacturer’s instructions. Appropriate dilutions were plated on 7H11 plates supplemented with OADC and CFU were counted after 21 days. For determination of neutrophil bacterial killing activity cells were infected with *M*. *smegmatis* or BCG at a MOI of 10. After 20 h of incubation, neutrophils were lyzed and bacterial CFU were determined after plating.

### Lung and bone marrow cell preparation, antibodies, flow cytometry and cell sorting

Lungs were perfused with 10 ml PBS. Single-cell suspensions were obtained from lung tissue after 1 h of treatment at 37°C with 1.5 mg/ml collagenase D and 40 units/ml DNAse A (Roche, Mannheim, Germany) in RPMI 1640 complete medium supplemented with 10% FBS (Gibco BRL, Life Technologies, Paisley, UK), 2 mM L-glutamine (Gibco), 100 U penicillin and 100 μg/ml streptomycin (Gibco). Cells were filtered through a nylon cell strainer with 100 μm pores (BD Falcon) and suspended in complete medium for stimulation with HK BCG. For the collection of cells after Broncho Alveolar Lavage (BAL), mice were killed and their lungs were washed four times with 0.5 ml PBS each time. The washing fluid from all four washes was pooled. BAL cells were washed once in PBS and were then suspended in complete medium. Bone marrow neutrophils were purified from bone-marrow by positive magnetic sorting (Miltenyi Biotec, Bergish Gladbach, Germany) after labeling with anti-Ly-6G PE-conjugated Ab (clone 1A8) as previously described [23]. For flow cytometry, cells were incubated for 20 min with 2% total mouse serum, and then with PBS supplemented with 5% FCS and 0.1% total mouse serum, for staining of the surface markers CD11b (clone M1/70), Ly6G (clone 1A8), Ly-6C (clone AL-21), CD3ε (clone 145-2C11), CD4 (clone L3T4), γδ (clone GL3) all from BD Biosciences (San Rose, CA, USA) Before intracellular staining for IFN-γ and IL-17A, T cells were incubated with 1 μg/ml ionomycin and 25 ng/ml M phorbol 12-myristate 13-acetate (PMA) (Sigma-Aldrich, Saint Louis, MO, USA) and HK BCG and treated for 4 h at 37°C with 5 μg/ml brefeldin A (Sigma-Aldrich). For the purification of lung neutrophils on days 1 and 23, CD11b^+^ Ly-6C^+^ Ly-6G^+^ cells were sorted on a MoFlo® high-speed cell sorter (Beckman Coulter, Fort Collins, CO, USA) with Summit Software (Beckman Coulter). Neutrophils were recovered in complete medium and immediately stimulated with HK-BCG.

### Detection of chemokines and cytokines by ELISA, measure of Reactive Oxygen Species and Myeloperoxidase activity

For the measurement of antigen-specific cytokine production by lung cells, we dispensed 10^5^ lung tissue cells in individual wells of 96-well plates. The cells were incubated at 37°C, under an atmosphere containing 5% CO_2_, and were stimulated with the equivalent of 10^6^ CFU of HK BCG. Supernatants were harvested 72 h later and assayed for IL-17A and IFN-**γ** by ELISA. CXCL-1, 2 and 5 production was measured in BAL fluids and lung tissue homogenates by ELISA. Commercial kits were from R&D Systems (Minneapolis, MN, USA)

Myeloperoxidase (MPO) activity was measured immediately after sorting and Reactive Oygen Species (ROS) produced by neutrophils were assessed after overnight stimulation with HK BCG (equivalent MOI of 10). 3x10^5^ neutrophils resuspended in 450 μl of PBS were lyzed by adding 450 μl of 0.05% Triton X100. After sonication and clearance by centrifugation for 10 min at 4°C and 12,000 x g. MPO was determined by mixing 30 μl of lysate with 70 μl of 3, 3’, 5, 5’-tetramethylbenzidine (Sigma Aldrich). After stopping the reaction with hydrochloric acid (Interchim, Montluçon, France), MPO activity was determined by measuring absorbance at 450 nm. ROS were measured in neutrophils after the addition of 10 μM ROS fluorescent detection reagent (Invitrogen) and incubation for 60 min at 37°C. Green fluorescence was read in a flow cytometer (FACS Calibur, BD Biosciences).

### Immunohistochemistry on mycobacteria-infected lungs

*In situ* lung neutrophil infection was analyzed in mice on day 1 or 23 after infection with 5x10^6^ CFU of the green fluorescent BCG strain Myc409 [7]. Freshly collected lungs were incubated overnight at 4°C in 4% paraformaldehyde, 10% sucrose (w/vol in PBS) and then immersed in 30% sucrose (w/vol in PBS) for 4 h. Lung tissue was snap frozen in OCT compound (CellPath, Powys, UK). 8 μm-thick lung sections collected on Superfrost Plus slides (Thermo Fischer Scientific) were air-dried and stored at -80°C. Cryosections were blocked for 30 min with Fc-blocking antibody 2.4 G2 (BD Biosciences), washed in PBS with 2% FCS and incubated overnight at 4°C with anti-Ly-6G (clone1A8), (BD Biosciences) followed by incubation with Alexa 594 conjugated anti-rat (Invitrogen) and anti CD3ε (145-2C11, APC conjugated, Miltenyi Biotec) for the localization of T cells and neutrophils. Slides were washed in PBS with 2% and mounted with Fluoromount-G (eBioscience) including DAPI. Images were captured with a confocal Leica TCS SP8 microscope.

### Statistical analysis

For analysis of *in vivo* data, results from two independent experiments were pooled. PRISM Graphpad 5.0 software (San Diego, CA, USA) was used for statistical analyses. We used the Kruskal-Wallis followed by Dunn’s *post hoc* test to analyze the kinetics of neutrophil recruitment to the lung and the nonparametric unpaired Mann-Whitney test for all other comparisons. (* *P* < 0.05, ** *P* < 0.01 and *** *P* < 0. 001).

## Results

### Neutrophils reach the lung in two waves following intranasal infection with attenuated BCG or virulent Mtb

**Fig 1:**
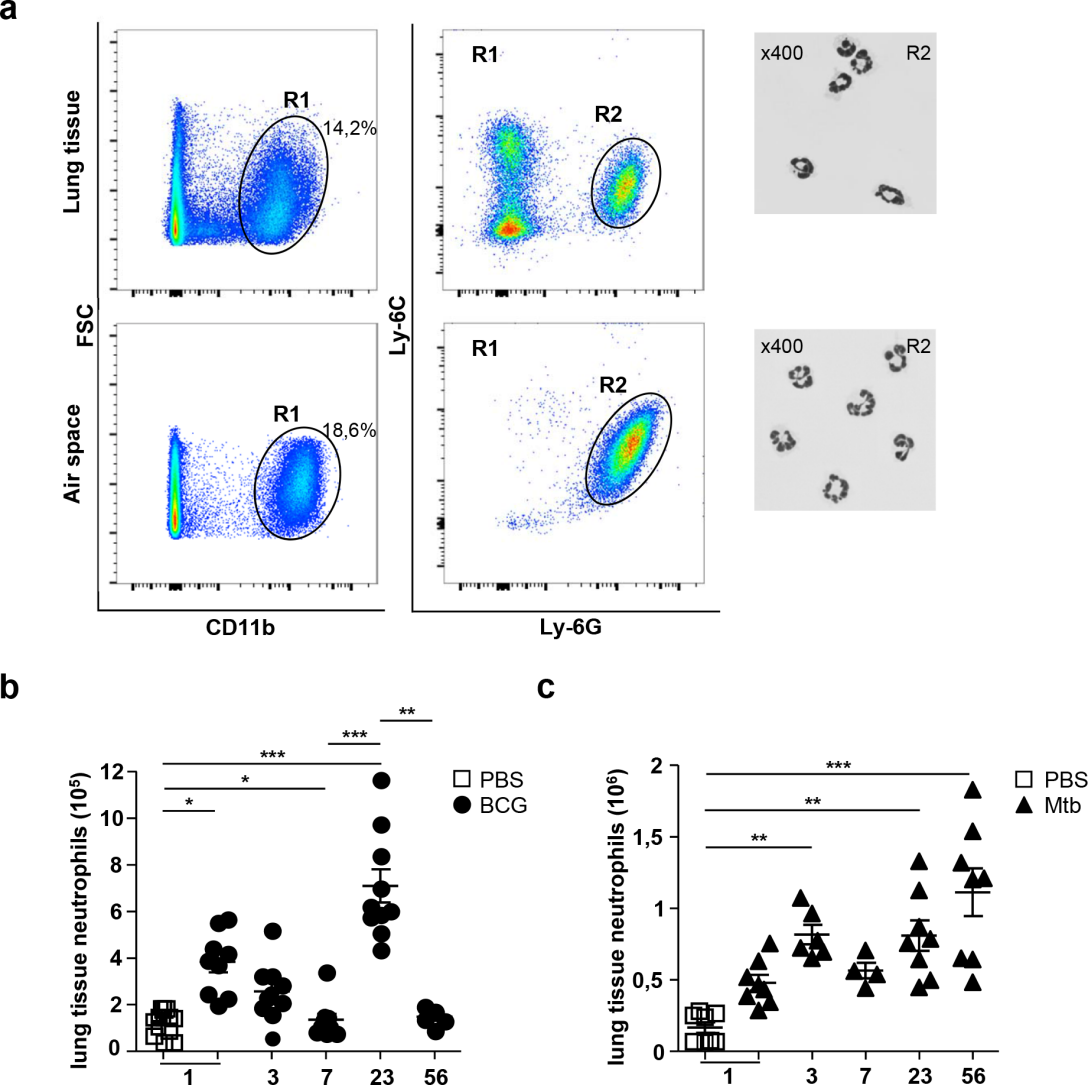
Neutrophils reach the lung in two waves following mycobacterial infection. Wild-type C57BL/6 mice were infected with 5x10^6^ CFU of BCG or 10^3^ CFU of Mtb and euthanized at the time points indicated. Cells were obtained from the lung tissue or airspace from individual animals. (**a**) Combined labeling with anti-CD11b, anti-Ly-6C and anti-Ly-6G antibodies was used to identify neutrophils among the cells present in the lung. The characteristic polylobed nucleus of neutrophils was observed in CD11b Ly-6C Ly-6G triple-positive cells from the R2 gate after flow cytometry sorting and May Grünwald Giemsa labeling. Slides were observed under an Eclipse 80 microscope (Nikon instrument) equipped with a 40 x objective. (**b, c**) We determined the numbers of neutrophils present in the lung tissue by flow cytometry for C57BL/6 mice killed on days 1, 3, 7, 23 and 56 post infection. Lung neutrophil numbers were determined after infection with BCG (**b**) or Mtb (**c**) Data from two independent experiments (n = 5–10). Kruskal-Wallis with Dunn’s *post hoc* test *P<0.05, ** *P*<0.01, *** *P*< 0.001.

We decided to investigate the kinetics of neutrophil recruitment to the lung in response to live attenuated BCG that is controlled by the host adaptive response, or virulent Mtb that is not. 5x10^6^ CFU of live attenuated BCG or 10^3^ CFU of virulent Mtb were administered i.n. to target the lung. Cells from the airspace, obtained after bronchoalveolar lavage (BAL), and from enzyme-digested lung tissue were analyzed by FACS. CD11b, Ly-6C and Ly-6G triple positive neutrophils, with characteristic polylobed nucleus (Fig 1A), were distinguished from inflammatory monocytes and myeloid-derived suppressor cells [24].

The day after BCG instillation, neutrophils were abundant in both lung tissue and airspace where they accounted for a high proportion of CD11b^+^ cells. They rapidly decreased after day 3. However, a second, larger peak of neutrophils was observed at day 23 (Fig 1B). In the airspace, neutrophils also appeared in two waves, at day 1 and 23 (S1 Fig). A kinetic study with additional time points showed that the second wave of neutrophil appeared in the lung between days 15 and 17 (S1 Fig). By day 56 after BCG instillation, the number of neutrophils declined (Fig 1B).

Following administration of 10^3^ CFU of virulent Mtb, we detected neutrophils in the lung tissue at day one, albeit in smaller numbers than in mice receiving high dose of BCG (Fig 1C). After a slight decrease at day 7, neutrophil numbers again increased by day 23. Neutrophils continued to accumulate in Mtb-infected lung tissue at day 56 (Fig 1C). Similar kinetics was observed in the airspace (S1 Fig).

**Fig 2:**
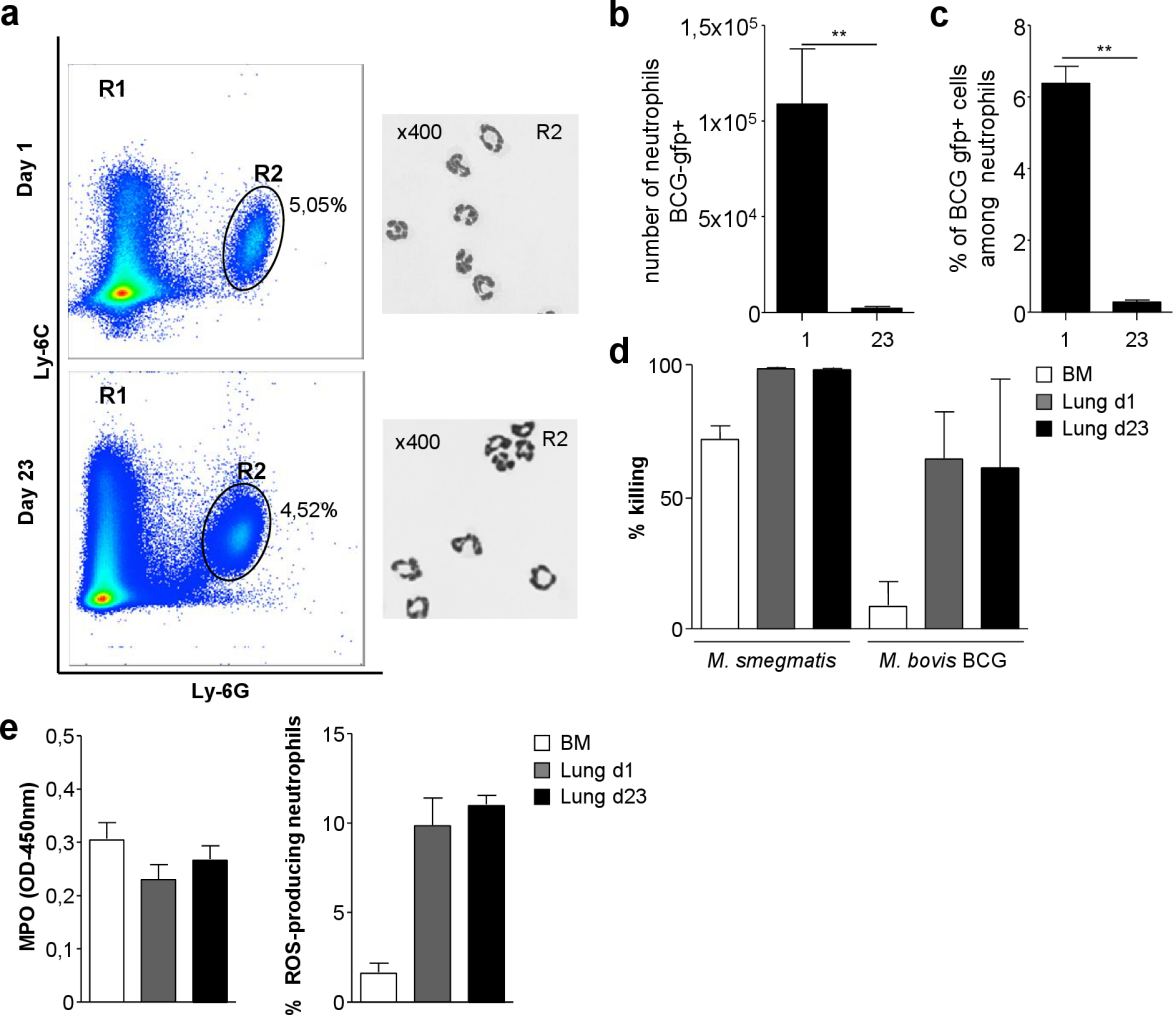
Day 1 or 23 lung neutrophils present similar killing abilities *ex vivo*. C57BL/6 mice were infected with 5x10^6^ CFU of BCG (**a, d, e**) or the green fluorescent recombinant BCG strain Myc 409 (**b, c**) (**a**) CD11b, Ly-6C, Ly-6G triple positive neutrophils recruited to the lung on day 1 or 23 were sorted by flow cytometry and stained with May Grünwald Giemsa. (**b)** Neutrophils carrying the green fluorescent BCG were analyzed by flow cytometry in five individual animals. Their numbers on day 1 and 23 are represented and mean +/-SD per lung. (**c**) In the same animals, the percentage of rBCG-*gfp*-infected, among all neutrophils are represented on day 1 or 23. (**d**) Lung neutrophils, sorted by flow cytometry at day 1 and 23 from BCG-infected mice or bone marrow neutrophils from naïve mice were infected with *M*. *smegmatis* or BCG at a MOI of 10. Bacterial killing was determined as the ratio of the initial inoculum to CFU obtained after overnight incubation with neutrophils, with 100% killing corresponding to no cell-associated bacilli. (**e**) The amounts of MPO and ROS bactericidal compounds produced by lung neutrophils purified at day 1 or 23 after BCG infection were compared with those produced by BM neutrophils. The results are shown as means ± SD for triplicate wells from two independent experiments (n = 6)

CD11b, Ly-6C, Ly-6G triple positive lung tissue neutrophils sorted by flow cytometry at day 1 and day 23 after BCG infection were then compared (Fig 2).

They displayed similar polymorphonuclear morphology (Fig 2A). In order to analyze their infection status *in vivo*, we used the green fluorescent BCG strain Myc409 [7]. Smaller numbers of BCG-infected neutrophils were recovered from lung tissue on day 23 than on day 1 (Fig 2B) despite a higher peak of recruitment (Fig 1B). Among all lung tissue neutrophils on day 1, 6% were infected with BCG whereas on day 23, less than 1% of neutrophils carried fluorescent bacilli (Fig 2C). Even though BCG phagocytosis by neutrophils seemed less frequent on day 23 than day 1, it remained detectable. Neutrophils were purified from the lung on day 1 or 23 after BCG instillation. Immature neutrophils were also purified from the bone-marrow for comparison. Cells were infected *in vitro* with BCG or the fast growing species *M*. *smegmatis*. After overnight incubation, intracellular bacterial CFU were determined and compared to the initial inoculum. Lung neutrophils were more effective than bone-marrow to eliminate bacilli. However lung neutrophils purified on day 1 or 23 displayed similar killing efficacy (Fig 2D). Myeloperoxidase (MPO) activity and reactive oxygen species (ROS) production by the different neutrophils was measured after *in vitro* BCG infection. All subsets displayed similar MPO activity (Fig 2E). Bone marrow neutrophils produced less ROS than lung tissue neutrophils. However lung neutrophils purified on day 1 or 23 post BCG infection did not display significant difference in ROS production (Fig 2E). Therefore, activated neutrophils recruited to the lung on day 1 or 23 after BCG infection were highly similar in phenotype, antimicrobial molecules production and killing ability.

### Neutrophils reaching the lung on day 23 do not control mycobacteria *in vivo*

**Fig 3:**
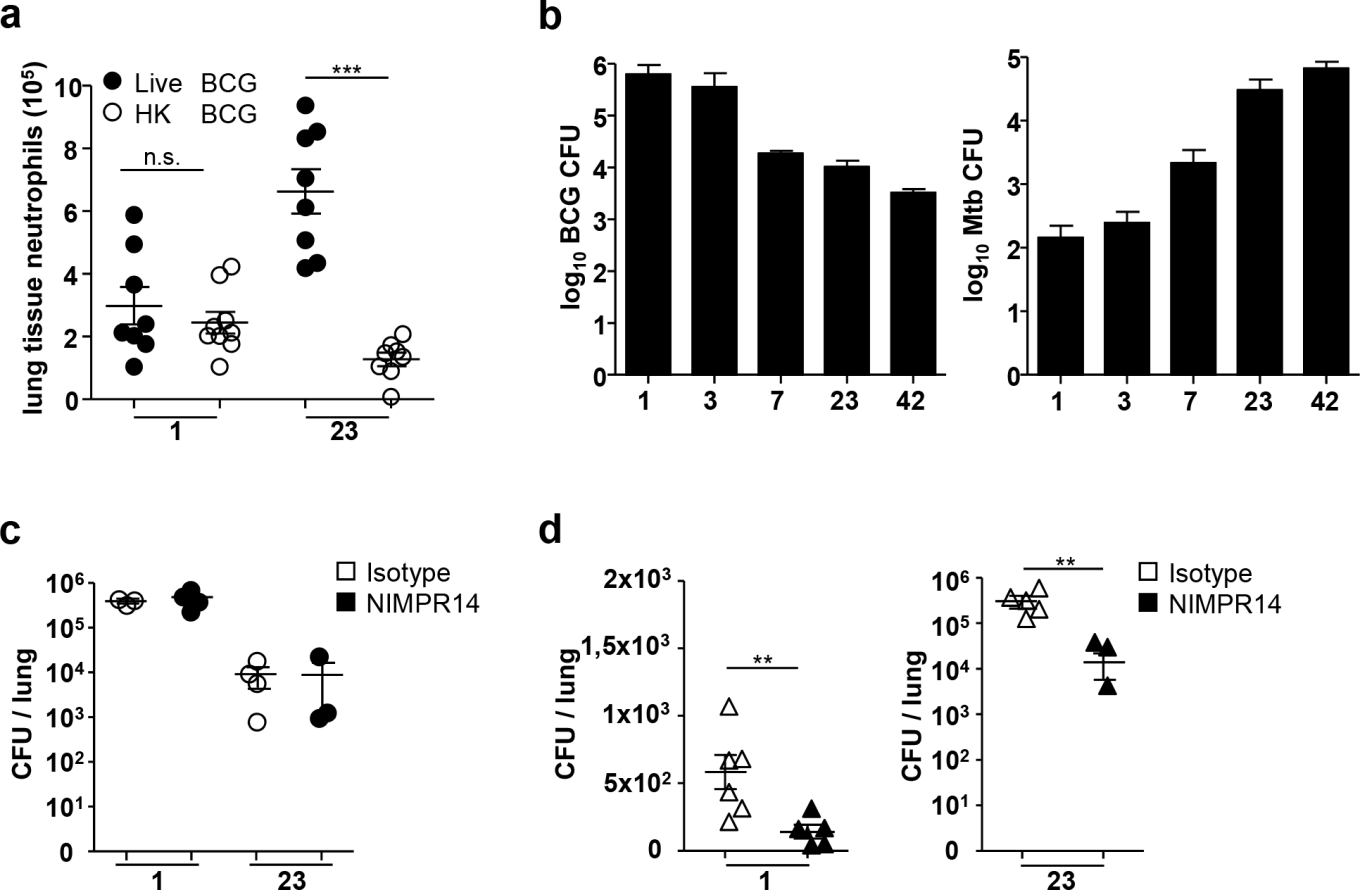
Neutrophils from the second wave do not kill mycobacteria *in vivo*. (**a**) Lung tissue neutrophil numbers from individual C57BL/6 mice were determined after flow cytometry analysis on day 1 and 23 after instillation with 5x10^6^ CFU of BCG or an equivalent load of heat-killed (HK) BCG. Data from two independent experiments (n = 8–9), ***P<0.001. (**b**) C57BL/6 mice (n = 5) were infected with 5x10^6^ CFU of BCG or 10^3^ CFU of Mtb. From day 1 to 42, the numbers of CFU in lungs were determined after bacterial growth on plates. (**c-d**) The impact of neutrophil depletion on bacterial control was measured by injecting mAb NIMPR14 or similar amounts of IgG2b control mAb into C57BL/6 mice 5, 3 and 1 day before infection with 5x10^6^ CFU of BCG (**c**) or 10^3^ CFU of Mtb (**d**) for the first neutrophil wave, or on day 17, 19 and 22 post infection for the second. The number of BCG or Mtb CFU present in the lung was determined on day 1 and 23 post infection. (**b-d**) The data shown are the mean ± SD of (log_10_) CFU counts from individual animals (n = 5–6), ** *P*<0.01

We then investigated the correlation between mycobacteria multiplication *in vivo* and the lung influx of neutrophils. One day after i.n. administration of 5x10^6^ CFU of live BCG or an equivalent load of heat-killed (HK) bacilli, similar numbers of neutrophils were recruited to the lung. By contrast, on day 23 the neutrophil peak was detected only in mice inoculated with live BCG (Fig 3A).

We then assessed the multiplication of attenuated BCG and virulent Mtb multiplication in the lung. As expected, by day 23 BCG CFU had decreased 100 times as compared to initial inoculum whereas virulent Mtb CFU had increased 100 times (Fig 3B). Therefore, the two waves of lung neutrophils were not directly correlated to the mycobacterial load. We then selectively depleted neutrophils on days 1 and 23 by injecting anti-Ly-6G NIMPR14 Ab (S1 Fig and [24]) either before mycobacterial infection or at days 17, 19 and 21 after infection. Neither of the neutrophil depletion had a measurable impact on BCG CFU in the lungs (Fig 3C). In Mtb infected mice, a moderate although significant decrease in CFU was observed when neutrophils were depleted in comparison to control mice (Fig 3D) indicating that the neutrophils had a negative impact on Mtb control. Therefore neutrophils reaching the lung on day 23 had no (BCG) or negative (Mtb) impact on mycobacterial multiplication *in situ*.

### Neutrophils from the second wave reach the lung at the same time as T cells with which they colocalize in the lung parenchyma

**Fig 4:**
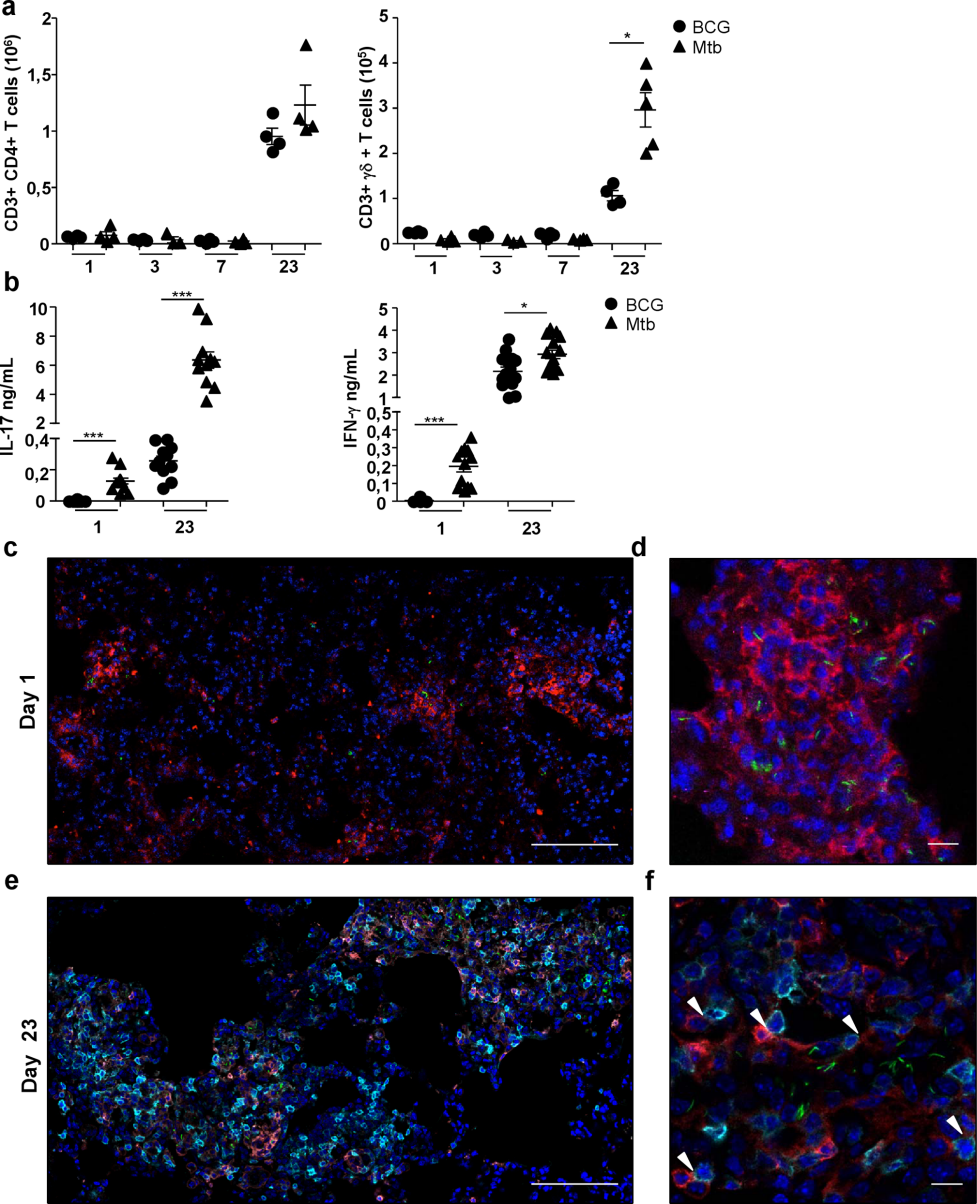
Neutrophils from the second wave reach the lung at the same time as effector T cells with which they establish intimate contacts. (**a**) Lung tissue cells from wild-type C57BL/6 mice, infected with 5x10^6^ CFU of BCG or 10^3^ CFU of Mtb, were labeled on day 1, 3, 7 and 23 and analyzed by flow cytometry. The numbers of CD3^+^ CD4^+^ conventional T cells or CD3^+^ γδ ^+^ T cells per mouse were determined (n = 4–5), *P<0.05. (**b**) On day 1 and 23 after infection with BCG or Mtb, 10^5^ total lung cells were cultured for three days in the presence of HK-BCG (equivalent to 10^6^ CFU). IL-17A and IFN-γ concentrations present in the supernatant were determined by ELISA (n = 8–10). Mann Whitney *P<0.05, ***P<0.001. (**c-f**) Representative confocal images of day 1 (**c, d**) or 23 (**e, f**) lung 8 μm cryosections from mice infected with green fluorescent BCG Myc 409 (green) and labeled with anti-Ly-6G for neutrophils (red), and anti-CD3 for T cells (cyan) and counterstained with DAPI for nuclei (blue). Merge triple or quadruple fluorescence of lung sections observed under low (**c, e**) or high magnification (**d, f**) showed neutrophil and BCG co localisation and absence of T cells on day 1 whereas neutrophils and T cell clusters were observed on day 23 (**e**). Higher-magnification (**f**) showed that on day 23, neutrophils barely co localized with mycobacteria and established close interactions with T cells (white arrowheads). Images were acquired with a confocal Leica TCS SP8 microscope equipped with x63 objective and were analyzed with the Leica LAS AF software.

We then analyzed T cell recruitment to the lung. Interestingly, coincident with the second peak of neutrophils, both lung CD3^+^ CD4^+^ αβ and CD3^+^ CD4^-^ γδ T cells peaked at day 23 in the BCG- and Mtb-infected mice (Fig 4A).

In a more detailed kinetic study, we observed that the second wave of neutrophils reached the lung at day 15 or 17 together with T cells (S1 Fig). CD3^+^ CD4^-^ γδ T cells were more numerous in Mtb than in BCG-infected mouse lungs (Fig 4A). As T-cell derived IFN-γ [12] and IL-17A [10] could be involved in the lung influx of neutrophils, we analyzed the production of these two cytokines in the lung. Both cytokines peaked at day 23 in total lung cells isolated from BCG or Mtb-infected mice (Fig 4B). IFN-γ production in response to Mtb infection was slightly higher than to BCG. By contrast, Il-17A production was 20 times more elevated in Mtb as compared to BCG infected mice indicating a more sustained inflammation in the first model. Expression of the *il-17a* gene but not the other genes from the *il-17* family [14] was up regulated in the lung at day 23 (S2 Fig). Consistent with previous reports [16–18], CD3^+^ CD4^+^ αβ T cells were the chief producers of IFN-γ, whereas CD3^+^ CD4^-^ γδ T cells produced most of the IL-17A (S2 Fig).

We then analyzed colocalization of neutrophils with BCG or T cells in lungs on day 1 or 23 following administration of the green fluorescent Myc409 strain. On day 1, the neutrophils mostly colocalized with BCG (Fig 4C and 4D), even though many bacilli were also observed inside Ly-6G negative cells, most probably alveolar macrophages (data not shown) and CD3^+^ T cells were not observed. By contrast, on day 23 the second wave of Ly-6G^+^ neutrophils mostly clustered with CD3^+^ T cells (Fig 4E) and the two cell types established close contacts (Fig 4F). At that time point BCG inside Ly-6G^+^ neutrophils were barely observed. Thus neutrophils that appeared in the lung on day 23 correlated with arrival of IFN-γ—and IL-17A-producing T cells in the lung but were not involved in direct mycobacterial control.

### IL-17RA on non-hematopoietic cells is necessary to control recruitment of neutrophils to the lung during the adaptive response

**Fig 5:**
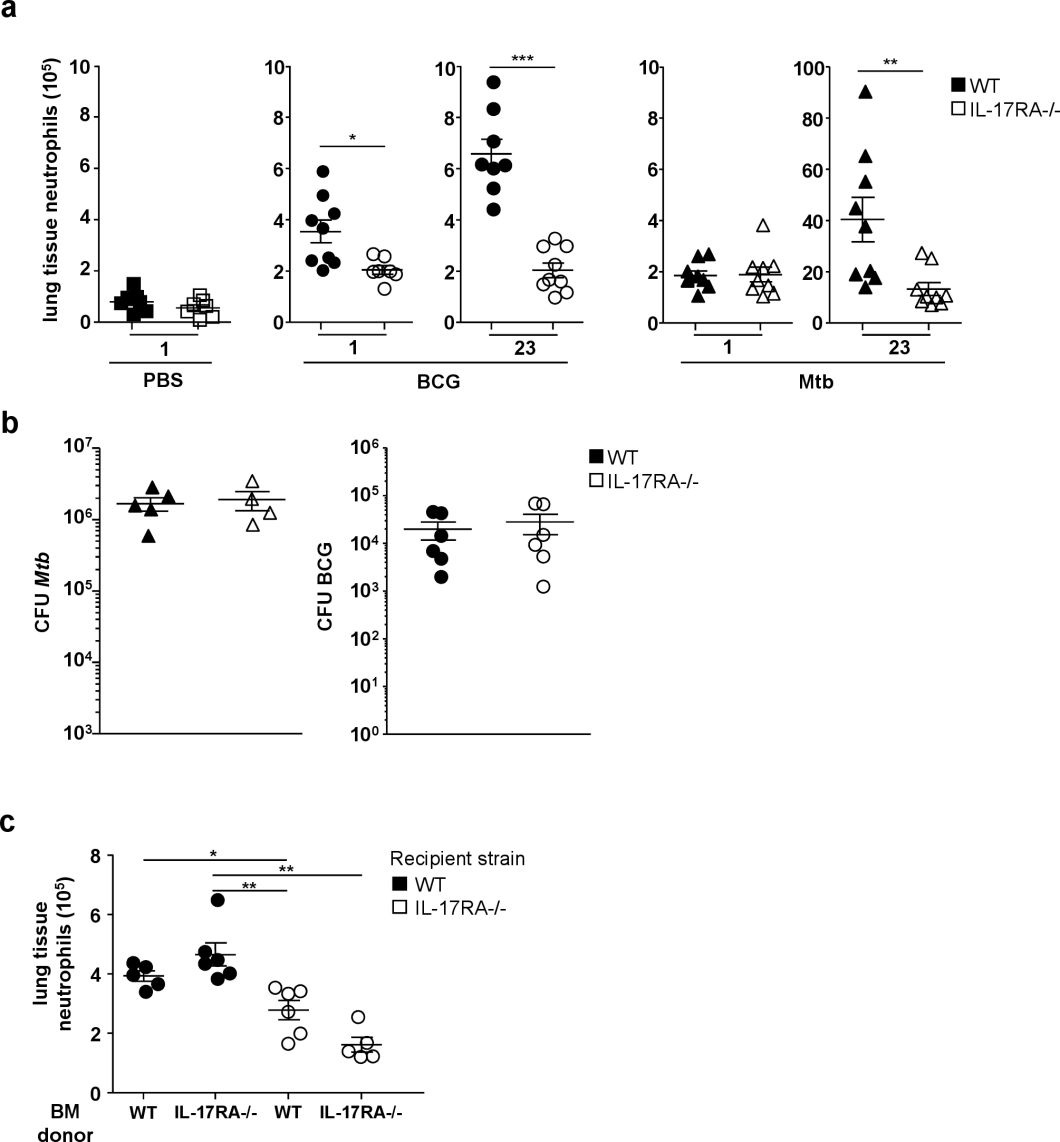
IL-17RA in non hematopoietic cells regulates recruitment of neutrophils to the lung during the adaptive phase of the immune response. (**a**) Neutrophils from C57BL/6 WT or IL-17RA^-/-^ mice infected with 5x10^6^ CFU of BCG or 10^3^ CFU of Mtb or mock-treated with PBS, were analyzed by flow cytometry the day following infection or 23 days later. Numbers of neutrophils per mouse (n = 7–9) were determined in lung tissues. *P<0.05, **P<0.01, ***P<0.001. (**b**) Number of CFU recovered from lungs from WT and IL-17RA^-/-^ mice (n = 5–6) 23 days after instillation of 5x10^6^ CFU of BCG or 10^3^ CFU of Mtb (**c**) Recipient WT and IL-17RA^-/-^ mice were lethally irradiated and reconstituted with 7x10^6^ bone marrow cells from WT or IL donor mice. On day 23 after infection with 5x10^6^ CFU of BCG, numbers of neutrophils recruited to the lung tissue cells was determined after flow cytometry analysis (n = 5–6) *P<0.05, *P<0.01.

Since IL-17A that peaked on day 23 is a key regulator of the mycobacterium granuloma [25], we decided to investigate neutrophil lung influx in IL-17RA^-/-^ mice [20], in which signaling by most members of the IL-17 family is blocked [26]. The day after BCG infection, neutrophils reached the lung tissue slightly less efficiently in IL-17RA^-/-^ than WT mice while in Mtb-infected mice, neutrophils reached the lungs in similar numbers whatever the genetic background (Fig 5A).

By contrast, on day 23 the influx of neutrophils to the lung tissue (Fig 5A) or airspace (data not shown) was severely impaired in Mtb or BCG-infected IL mice. The mycobacterial load was similar in WT and IL-17RA^-/-^ mice 23 days after BCG or Mtb infection (Fig 5B) showing again that neutrophils on day 23 did not play a major role in mycobacterial control (Fig 5B).

Since IL-17RA is ubiquitously expressed, we decided to investigate hematopoietic and non-hematopoietic IL-17RA contribution to neutrophil recruitment. To this end, lethally irradiated recipient WT or IL-17RA^-/-^ mice were reconstituted with donor WT or IL-17RA^-/-^ BM cells. All cells from control WT→WT mice expressed IL-17RA whereas IL-17RA^-/-^→ IL-17RA^-/-^mice were totally IL-17RA-deficient. Reconstitution was complete after six weeks (data not shown) and the mice were inoculated i.n. with 5x10^6^ CFU of BCG. Twenty-three days later, we analyzed the adaptive neutrophil recruitment in lung tissue and airspace. WT mice reconstituted with IL-17RA^-/-^ BM cells (IL-17RA^-/-^→WT) displayed lung tissue (Fig 5C) or airspace (data not shown) neutrophil numbers similar to control WT→WT mice. By contrast, adaptive lung neutrophil recruitment was severely impaired in WT→ IL-17RA^-/-^ as in IL-17RA^-/-^→ IL-17RA^-/-^ mice where IL-17RA-/- expression by non hematopoietic cells was abrogated (Fig 5C).

### IL-17RA regulates CXL-1 and 5 production in the lungs during the adaptive response

**Fig 6:**
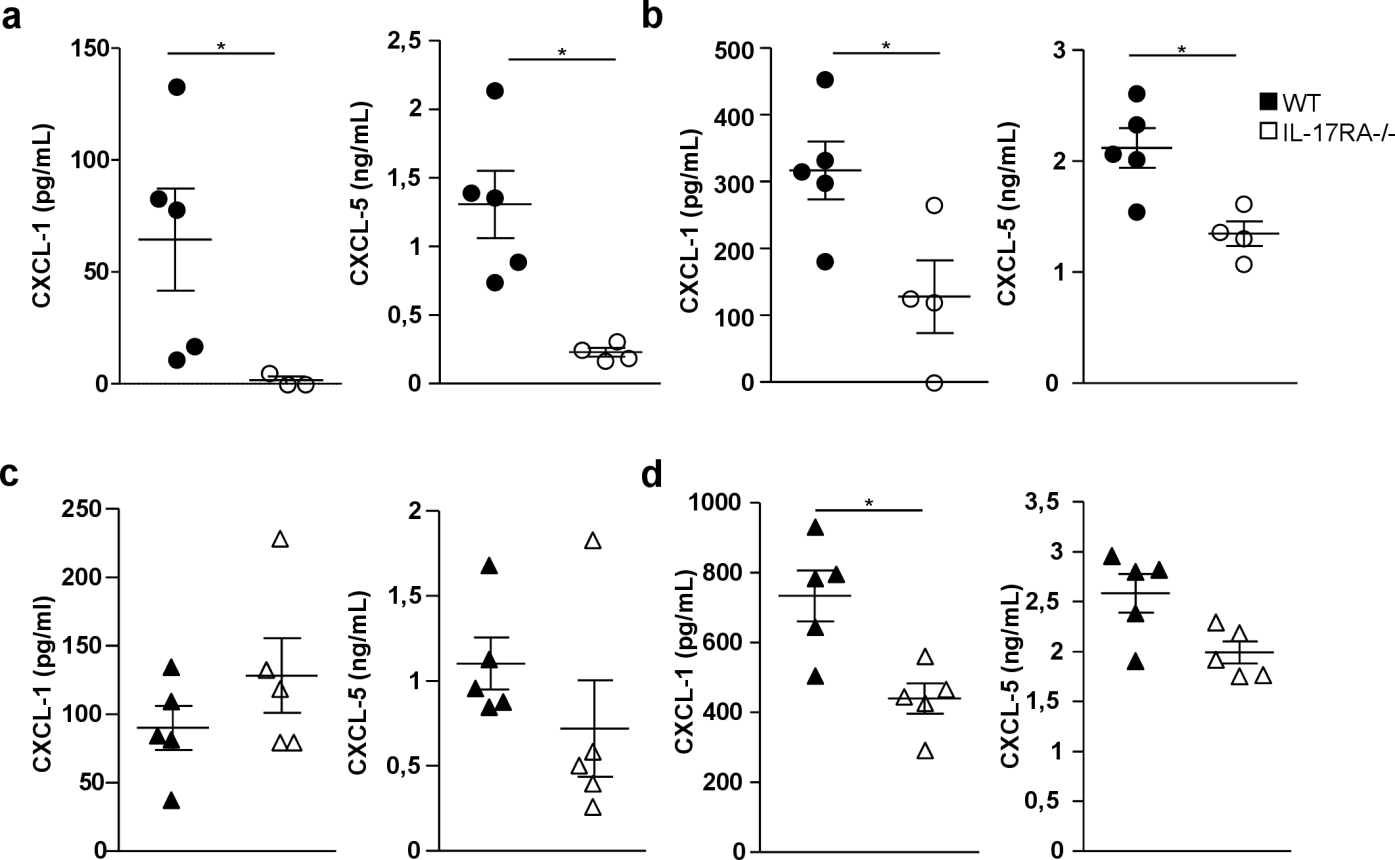
IL-17RA controls the lung production of CXCL-1 and 5 after BCG or Mtb infection WT or IL-17RA^-/-^ mice were infected with 5x10^6^ CFU of BCG (**a, b**) or 10^3^ CFU of Mtb (**c, d**) and production of CXCL-1 and 5 was analyzed by ELISA in BAL fluid (**A, C**) or lung tissue homogenates (**b, d**) on and 23 infection. Data from n = 4–5 animals are represented. *P<0.05.

CXCL-1 (KC), CXCL-2 (MIP2) and CXCL-5 (LIX) are three main CXCR2 ligands involved in neutrophil recruitment [27]. We next investigated whether IL-17RA controlled the production of these chemokines in the lungs BCG or Mtb-infected WT and IL-17RA^-/-^ mice. On day 23 after infection, ELISA was performed on the BAL fluids and cleared lung tissue homogenates to measure the three chemokines. CXCL-2 was detected in lung homogenates only and did not differ between WT and IL-17RA^-/-^ mice (data not shown). In BCG infected mice, we observed an impaired production of both CXCL-1 and 5 in IL-17RA^-/-^ mice as compared to WT (Fig 6A and 6B).

The same trend was observed in Mtb infected animals although it reached the statistical level only for CXCL-1 in lung tissue homogenates (Fig 6C and 6D). Therefore IL-17RA expression in the lungs was required for optimal production of two of the three main CXCR2 ligands i.e. CXCL-1 and 5 after lung infection with BCG. The same trend was observed for Mtb.

### CXCL-1 and 5 instillation recapitulates the neutrophil recruitment default in lungs of IL-17RA-/- mice during the adaptive response

**Fig 7:**
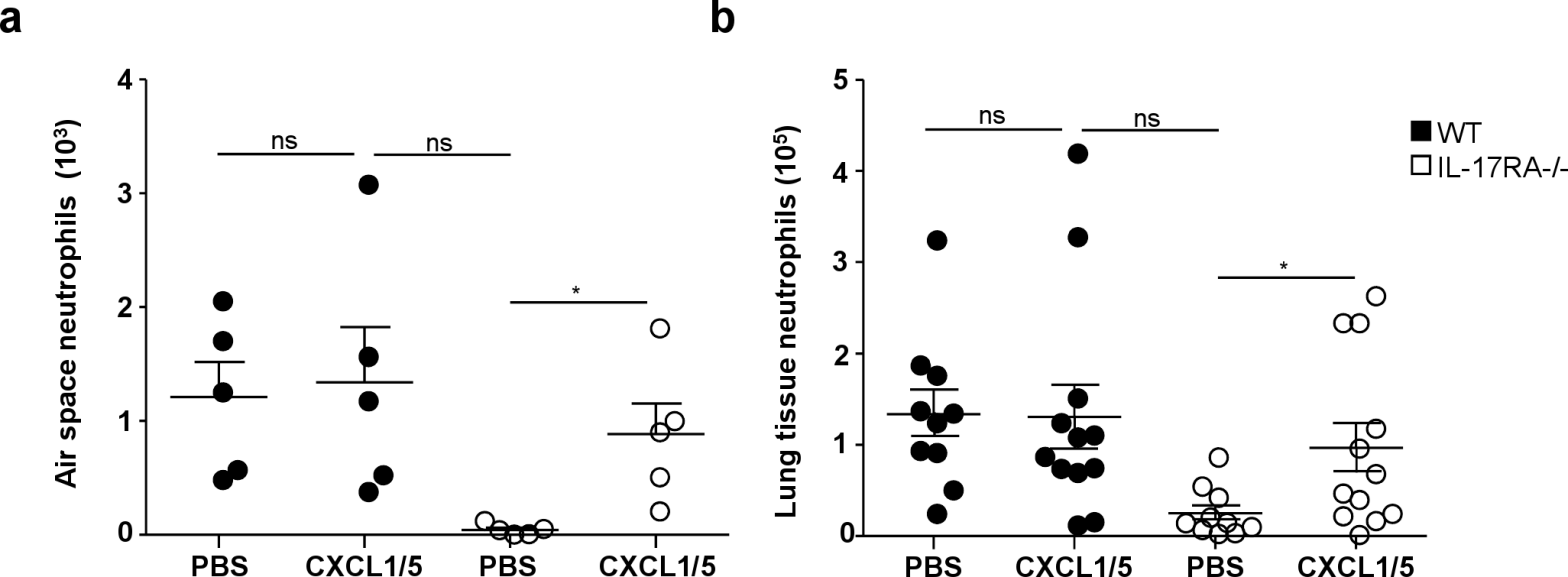
CXCL-1 and 5 intranasal inoculation restores neutrophil recruitment to the lungs of BCG-infected IL-17RA-/- mice WT or IL-17RA-/- mice were infected with 5x106 CFU of BCG. On day 20 after infection, a mixture of recombinant CXCL-1 and 5 in PBS, 100 μg each, was instilled into 5 animals while controls received PBS only. On day 23, neutrophils recruited into the airspace (**a**) or lung tissue (**b**) were analyzed by flow cytometry. Numbers of neutrophils present in 5 BAL pooled from 2 to 3 mice (**a**) or in lung tissue from 10 to 12 individual animals from two independent experiments are depicted (**b**). * *P*<0.05

We next decided to assess if the default of CXCL-1 and 5 production in IL-17RA^-/-^ mice during the adaptive phase of the immune response to mycobacterial infection was responsible for the impaired recruitment of neutrophils to the lung. WT and IL-17RA^-/-^ mice were infected with BCG and on day 20 they received an instillation of a mixture of 100 μg of each of the two chemokines CXCL-1 and 5. Control animals received a similar volume of PBS. Three days later CD11b, Ly-6C, Ly-6G triple positive neutrophils recruited to the airspace and lung tissue were analyzed by flow cytometry and counted. In WT mice similar numbers of neutrophils were observed into the air space (Fig 7A) and lung tissue (Fig 7B) after instillation of PBS or the two chemokines.

By marked contrast, in IL-17RA^-/-^ mice, instillation of CXCL-1 and 5, and not PBS, allowed recruitment of neutrophils to the air space (Fig 7A) and the lung tissue (Fig 7B) with levels comparable to WT animals. Therefore, the defective neutrophil recruitment to the lung observed in IL-17RA^-/-^ mice during the onset of the adaptive response after BCG infection, could be restored by local introduction of CXCL-1 and 5.

## Discussion

In response to mycobacterial infection, neutrophils may exert protective or deleterious functions [28, 29]. In order to gain more insight into pathways that control neutrophil recruitment to the lung during mycobacterial infection, we decided to use two different models. We instilled into the lung either a high dose of the live attenuated strain BCG, that induced a high early inflammatory response and was later controlled by the host immune response, or a low dose of fully virulent Mtb that induced less initial inflammation but displayed unrestricted multiplication. In these two opposite situations we observed that neutrophil recruitment to the lung was biphasic. In addition to the swift recruitment of neutrophils during the first 24 hours after bacterial entry into the lung, a second wave of neutrophils reached the lung at the same time as primed T cells. These data, obtained after Mtb or BCG administration targeting the natural site of infection, i.e. the lung are in agreement with pioneering work carried out by Appelberg and coworkers who showed T cell-dependent chronic neutrophilia in the peritoneum of *M*. *avium* or Mtb infected mice [30–32]. However our data differ regarding the role played by these T-cell dependent neutrophils. While Pedrosa *et al* [33] reported that neutrophils played a protective role, in our case T-cell dependent neutrophils had no measurable—for BCG—or negative—for Mtb—impact (our data and [34]) on bacilli growth *in vivo*, despite *in vitro* killing ability. We think that this discrepancy is mostly due to differences in inoculation routes and to the antibody RB6-8C5 that was used by Pedrosa *et al* that is now established to deplete other cells in addition to neutrophils [35]. Instillation of HK BCG resulted in a barely detectable second wave of neutrophil recruitment, indicating that the persistence of mycobacteria, inducing a sustained protective immune response [36, 37], was required for this second wave. Furthermore, they barely co localized with bacteria in the lung, and built up intimate contacts with T cells. These observations emphasize the ability of this second wave of neutrophils to take part to the adaptive immune response and we propose to define them as “adaptive neutrophils”.

We also established that the IL-17A/F-IL-17RA pathway was a key regulator of the adaptive wave of neutrophil recruitment to lungs infected with Mtb or BCG. This pathway was less important for the influx of early neutrophils. The signals involved in early neutrophil recruitment to the lung remain to be established, but it is possible that several compounds from the mycobacterial cell wall, recognized by neutrophil-expressed pathogen recognition receptors, are directly involved [38]. The IL-17RA-driven neutrophil recruitment pathway was rather connected to the onset of the adaptive T cell response, whether it was induced by an attenuated or a virulent strain.

IL-17RA is ubiquitously expressed [14] and most cells are therefore potentially able to respond to IL-17A. However, we established, in studies on BM-chimeric mice, that IL-17RA expression by non-hematopoietic cells controlled the recruitment of adaptive neutrophils. In response to IL-17A or F, the lung epithelium produces CXCR2 ligands, such as CXCL-1 (KC), CXCL-2 (MIP2) and CXCL-5 (LIX), which act as powerful attractants of neutrophils [39]. In the lung, CXCL5 is the principal ligand responsible for sustained neutrophil attraction after Mtb infection [40]. In BCG infected mice we observed that, at the time of adaptive neutrophil recruitment to the lung, production of CXCL-1 and 5 was impaired in IL-17RA deficient mice as compared to WT. Interestingly, production of CXCL-2, the third main ligand for CXCR2, was observed in presence or absence of IL-17RA. A similar trend was also observed in Mtb-infected mice although it did not reach statistical significance. Despite similar CXCL-2 production in IL-17RA-/- and WT mice, recruitment of adaptive neutrophils was highly impaired in IL-17RA^-/-^ mouse lungs indicating that CXCL-1 and 5 were the most important. This was confirmed after local instillation of CXCL-1 and 5 that restored adaptive neutrophils recruitment in IL-17RA-/- BCG-infected mice. The three ligands may have different affinities for CXCR2 expressed by adaptive neutrophils and CXCL-2 may not be well recognized. Alternatively, CXCL-1 or 5, but not 2, may signal via a neutrophil chemokine receptor different from CXCR2 specifically expressed by adaptive neutrophils. Although we cannot exclude that other chemokines such as CXCL-3, 6, 7 and 8, signaling via CXCR1 or 2 in humans [27], or CXCL15/lungkine [41] are involved in neutrophil recruitment during the adaptive phase of the immune response, our data indicate that CXCL-1 and 5 are the chief drivers.

Several reports have highlighted the importance of IL-17A production in the control of mycobacterial infection. Memory Th17 cells induced by vaccination mediate an optimal Th1 protective response against Mtb in the mouse lung [15] and IL-17A is critically involved in mature granuloma formation, which helps to constrain mycobacterial multiplication [17, 18] and is required to restrict multiplication of the hypervirulent Mtb strain HN878 [19]. We observed no difference in the control of BCG or lab adapted Mtb H37Rv multiplication between WT and IL-17RA-/- mice, from day 1 to 56, in agreement with previous data obtained after short term low-dose infection with Mtb [42]. However, IL-17RA might play an important role in longer terms [43] i.e. when the Mtb bacilli load becomes more important. Nevertheless, the IL-17A-driven exacerbation of inflammation observed in several circumstances may lead to tissue destruction. IL-17A is overproduced in response to high mycobacterial loads in the mouse [10] or in humans infected with multidrug-resistant Mtb strains resulting in high antigen loads [44], leading to an unfavorable clinical outcome. The IL-17A/IL-17RA pathway thus appears to be something of a double-edged sword in the control of Mtb-induced disease. We observed that during the adaptive response, neutrophils did not play an important role in mycobacterial control. However, neutrophils were recruited to the lung at the same time as T cells with which they established intimate contacts, suggesting their potential role in T cell regulation. Indeed we have previously shown *in vitro* that neutrophils, when in contact with BCG-infected dendritic cells and T cells, were able to produce IL-10 that specifically shut down Th17 CD4 T cells through their IL-10 receptor [23]. Such immunosuppressive IL-10 producing neutrophils were also described *in vivo*, in the lung of Mtb-infected mice by Zhang *et al* [34] where they had a negative impact on Mtb control as we observed in our experiments.

Whether neutrophils have positive or negative impact in TB in humans remains a matter of debate. Whatever their role, these cells represent an important target for the development of new host-directed therapies to combat TB that are urgently needed to combat multidrug resistant strains [45]. In that respect our findings in the mouse model indicate that the IL-17RA-driven production of CXCL-1 and 5 in the lung is a new research axis to be considered.

## Supporting Information

S1 Appendix Assessment of IL-17A gene expression by RT-qPCR. (PDF)

S1 Fig Kinetics of cell recruitment to the lung after BCG or Mtb infection. C57BL/6 WT mice were inoculated by the intranasal route with 5x10^6^ CFUs of BCG (**all panels except b**) or 10^3^ CFUs of Mtb (**b**) and euthanized on indicated days. Cells from the airspace recovered after bronchoalveloar lavage (**a, b**) or enzyme lung-tissue digestion (**c, e**) were analyzed by flow cytometry with antibodies, as indicated in materials and methods. CD11b^+^ Ly-6C^+^ Ly-6G^+^ neutrophils (**a-d**) and CD3^+^ CD4^+^ γδ^-^ and CD3^+^ CD4^-^ γδ^+^ T cells (**e**) from four independent mice were counted *P<0.05. (**d**) Three injections of NIMP-R14 Ab or isotype control were administered i.p to mice on days 17, 20 and 22 after BCG-infection. Neutrophils present in lung tissue were numerated on day 23 (n = 6) **P< 0,005.
(PDF)

S2 Fig IL-17 family genes expression in lungs from BCG or Mtb infected mice and IL-17A production by lung T cell subtypes. C57BL/6 mice were infected i.n. with 5x10^6^ CFUs of BCG or 10^3^ CFUs of Mtb or treated with PBS. (**a**) Lungs were harvested on day 1 or 23 and processed for mRNA isolation and cDNA preparation. Quantitative real-time PCR was performed on the cDNA as described in supporting materials and methods. The expression of the *il-17 a*, *c*, *e* and *f* genes was normalized with respect to the mean expression levels of three housekeeping genes: *hrprt*-1, *rel*-4 and *ppia*. The data shown are means ± SD of four to five individual mice. The only gene displaying significant upregulation was *il-17a*, which was more strongly expressed on day 23 than on day 1, with similar levels of expression in BCG- and Mtb-infected mice. (**b**) Lung cells from day 23 BCG-infected mice were stimulated by incubation overnight with HK BCG and PMA ionomycin and then stained for intracellular IL-17A and IFN-γ. Among the CD3^+^ T cells, CD4^+^ γδ^-^ T cells were the main producers of IFN-γ, whereas CD3^+^ γδ^+^ T cells mainly produced IL-17A. The data shown are the mean ± SD percentages of positive cells from duplicate wells.
(PDF)
