# Supplementary material for: IL-17RA in Non-Hematopoietic Cells Controls CXCL-1 and 5 Critical to Recruit Neutrophils to the Lung of Mycobacteria-Infected Mice during the Adaptive Immune Response
Source: PLoS One. 2016 Feb 12;11(2):e0149455. doi: 10.1371/journal.pone.0149455 (PMC4752258; doi:10.1371/journal.pone.0149455)
Supplement: S1 Appendix — (PDF) [file pone.0149455.s001.pdf]

S1 Appendix Assessment of IL-17A gene expression by RT-qPCR

Lung tissues were perfused with PBS and disrupted in 1 ml Trizol reagent (Sigma-Aldrich) with ceramic beads (Lysing Matrix D, MP Biomedicals) in a Ribolyser apparatus (Fastprep-24, MP Biomedicals). mRNA were extracted with the NucleoSpin RNAII kit (Macherey Nagel, Düren, Germany). RNA integrity was assessed with an Agilent RNA 6000 bioanalyzer (Agilent Technologies, Santa Clara, CA). After retro transcription with the M-MLV reverse transcriptase (Superscript III kit, Invitrogen, Carlsbad, CA, USA), real-time PCR was performed in a volume of 12.5 µl per well, with MESA Green (MasterMix Plus for SYBR® Assay, Eurogentec, Seraing, Belgium) in a Chromo 4 light cycler apparatus (Biorad, Hercules, CA, USA). Primers (Eurogentec) with the following sequences were used : *hprt*-1 F, 5'-GAGCGTTGGGCTTACCTCAC-3' ; *hprt*-1 R, 5'-GATGGCCTCCCATCTCCTTC-3' ; *ppia* F, 5'-CCACCGTGTTCTTCGACATC-3' ; *ppia* R, 5'-CCAGGACCTGTATGCTTTAG-3' ; *rpl-4* F, 5'-TCACTTTGCCAGCTGTGTTC-3' ; *rpl-4* R, 5'-GAACAGCCTCCTTGGTCTTC-3' ; *il-17a* F, 5'-CAAACACTGAGGCCAAGGAC-3' ; *il-17a* R, 5'-CACTGAGCTTCCCAGATCAC-3' ; *il-17c* F, 5'-AGGACCTCTAGCTGGAACAC-3' ; *il-17c* R, 5'-TGGGTAGCGGTTCTCATCTG-3' ; *il-17e* F, 5'-CCCAGCAAAGAGCAAGAACC-3' ; *il-17e* R, 5'-ACCCGATTCAAGTCCCTGTC-3' ; *il-17f* F, 5'-AAACCAGGGCATTTCTGTCC-3' ; *il-17f* R, 5'-ATTGATGCAGCCTGAGTGTC-3' ; *cxcl-1* F, 5'-CCAACACAGCACCATGATCC-3' ; *cxcl-1* R, 5'-TGAGGGCAACACCTTCAAGC-3' ; *cxcl-2* F, 5'-CACCAACCACCAGGCTACAG-3' ; *cxcl-2* R, 5'-CGCCCTTGAGAGTGGCTATG-3' ; *cxcl-5* F, 5'-TTCCATCTCGCCATTCATGC-3' ; *cxcl-5* R, 5'-GGGATCACCTCCAAATTAGC-3'. Gene expression was expressed relative to the mean level of expression for three housekeeping genes — hypoxanthine phosphoribosyltransferase

1(*Hprt-1*), ribosomal protein L4 (*Rpl-4*) and peptidylprolyl isomerase A (*Ppia*) — analyzed with GenEx software (Multid Analysis, Gothenburg, Sweden).
