# Supplementary figures and images for: IL-17RA in Non-Hematopoietic Cells Controls CXCL-1 and 5 Critical to Recruit Neutrophils to the Lung of Mycobacteria-Infected Mice during the Adaptive Immune Response
Source: PLoS One. 2016 Feb 12;11(2):e0149455. doi: 10.1371/journal.pone.0149455 (PMC4752258; doi:10.1371/journal.pone.0149455)

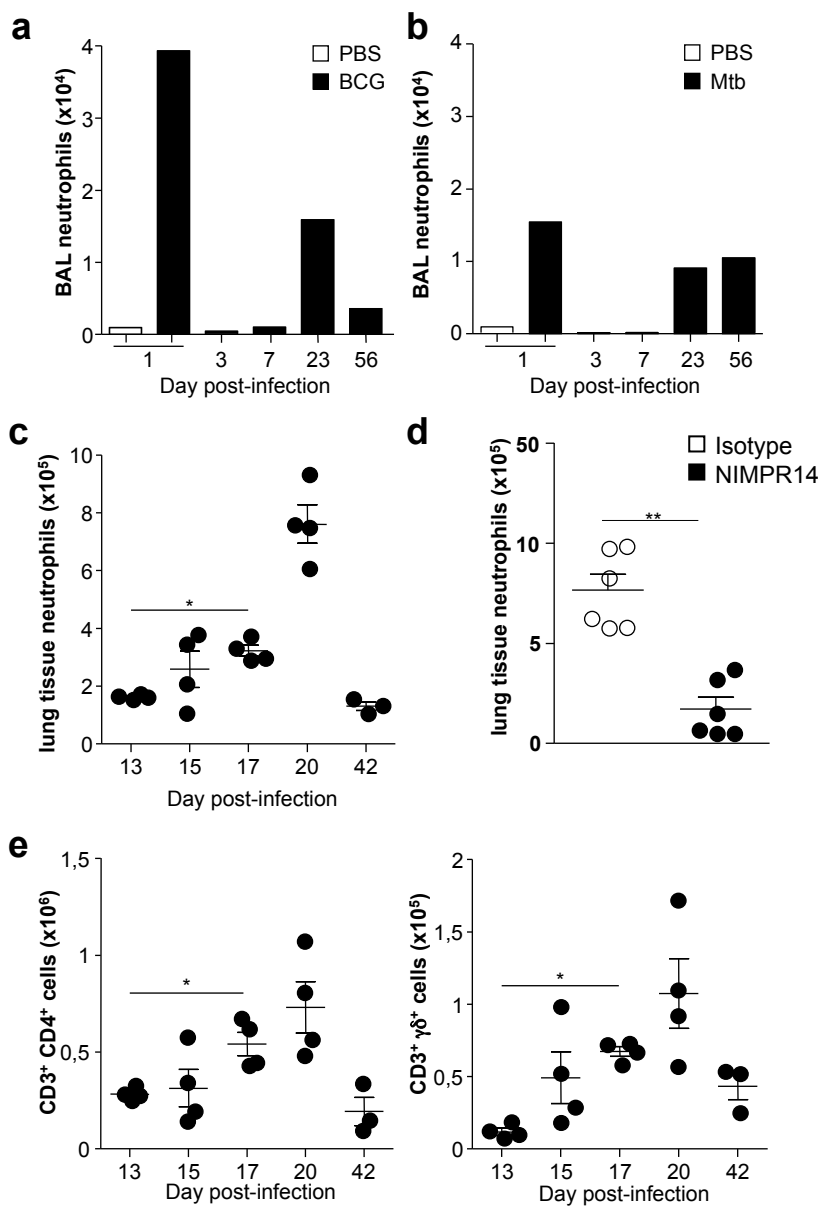

Supplement: S1 Fig — C57BL/6 WT mice were inoculated by the intranasal route with 5x10^6^ CFUs of BCG (all panels except b) or 103 CFUs of Mtb (b) and euthanized on indicated days. Cells from the airspace recovered after bronchoalveloar lavage (a, b) or enzyme lung-tissue digestion (c, e) were analyzed by flow cytometry with antibodies, as indicated in materials and methods. CD11b+ Ly-6C+ Ly-6G+ neutrophils (a-d) and CD3+ CD4+ γδ- and CD3+ CD4- γδ+ T cells (e) from four independent mice were counted *P<0.05. (d) Three injections of NIMP-R14 Ab or isotype control were administered i.p to mice on days 17, 20 and 22 after BCG-infection. Neutrophils present in lung tissue were numerated on day 23 (n = 6) **P< 0,005. (PDF) [file pone.0149455.s002.pdf]

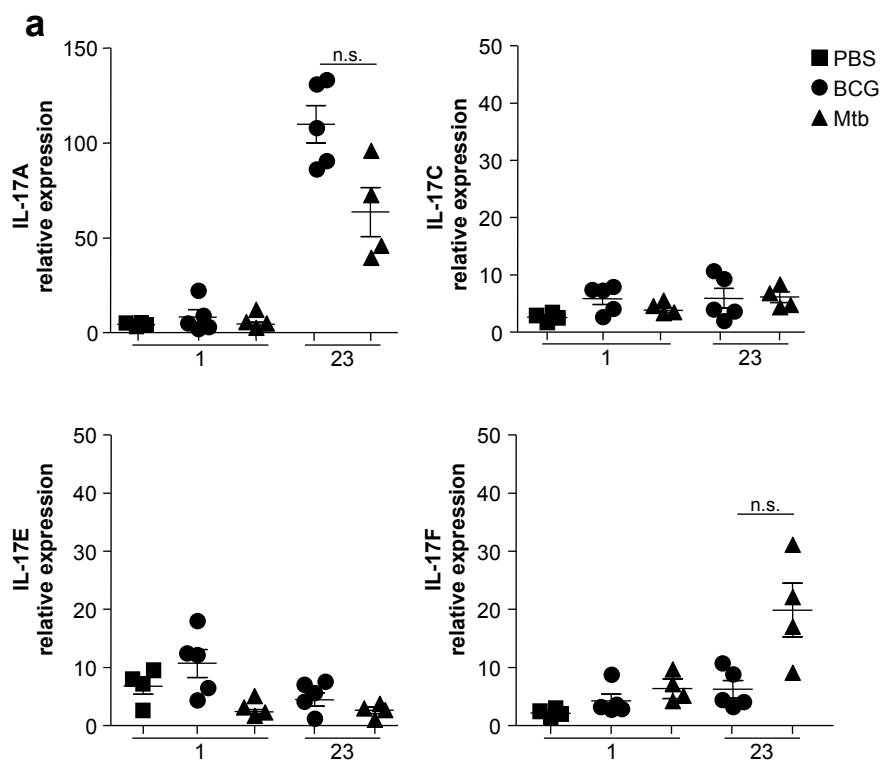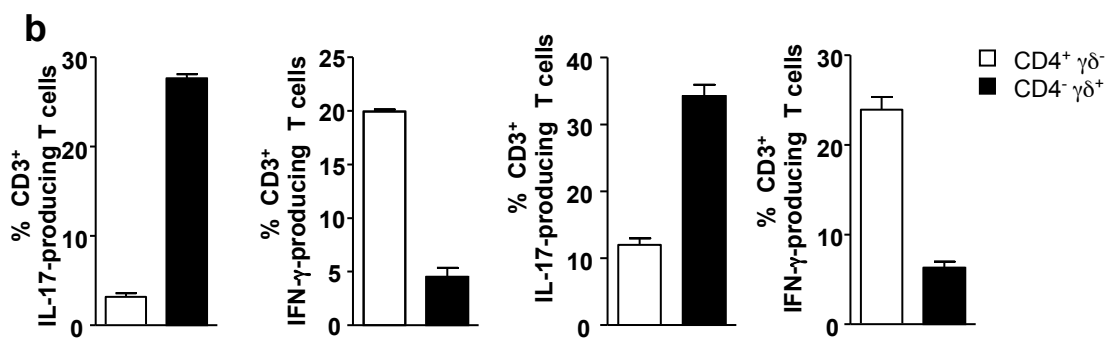

Supplement: S2 Fig — C57BL/6 mice were infected i.n. with 5x10^6^ CFUs of BCG or 103 CFUs of Mtb or treated with PBS. (a) Lungs were harvested on day 1 or 23 and processed for mRNA isolation and cDNA preparation. Quantitative real-time PCR was performed on the cDNA as described in supporting materials and methods. The expression of the il-17 a, c, e and f genes was normalized with respect to the mean expression levels of three housekeeping genes: hrprt-1, rel-4 and ppia. The data shown are means ± SD of four to five individual mice. The only gene displaying significant upregulation was il-17a, which was more strongly expressed on day 23 than on day 1, with similar levels of expression in BCG- and Mtb-infected mice. (b) Lung cells from day 23 BCG-infected mice were stimulated by incubation overnight with HK BCG and PMA ionomycin and then stained for intracellular IL-17A and IFN-γ. Among the CD3+ T cells, CD4+ γδ- T cells were the main producers of IFN-γ, whereas CD3+ γδ+ T cells mainly produced IL-17A. The data shown are the mean ± SD percentages of positive cells from duplicate wells. (PDF) [file pone.0149455.s003.pdf]
